# Tofacitinib, two-faced Janus in ulcerative colitis and Crohn’s disease?

**DOI:** 10.1177/2050640620942641

**Published:** 2020-07-08

**Authors:** Vince BC Biemans, Bram Verstockt

**Affiliations:** 1 Department of Gastroenterology and Hepatology, Radboud University Medical Centre, Nijmegen, The Netherlands; 2 Department of Gastroenterology and Hepatology, Maastricht University Medical Center, Maastrict, The Netherlands; 3 Department of Gastroenterology and Hepatology, University Hospitals Leuven, Leuven, Belgium; 4 Department of Chronic Diseases, Metabolism and Ageing, KU Leuven, Leuven, Belgium

Tofacitinib, an oral small molecule, was recently added to the rapidly evolving therapeutic landscape of ulcerative colitis (UC), as a first-in-class pan-Janus kinases (JAK) inhibitor. Because of the increasing number of licensed compounds, with even more agents in late stage development,^1^ questions on the current and future place of tofacitinib in inflammatory bowel disease become highly relevant.

In comparison with monoclonal antibodies, tofacitinib clearly has a few unique characteristics and interesting advantages, including its oral administration, rapid onset of action, quick clearance and lack of immunogenicity, as summarised by Magro and Estevinho.^2^ However, by acting on multiple cytokine signalling pathways,^3^ its broad immunosuppressive features, interference with lipid metabolism and increased risk of venous thromboembolism warrants caution. Although efficacy increases with higher tofacitinib exposure, adverse events are also dose-dependent and thus high dosing (10 mg twice a day (BID)) should be limited in time. Selective JAK inhibitors, including filgotinib (SELECTION phase 2b/3 programme, ClinicalTrials.gov identifier: NCT02914522) are currently being tested in UC and show clear efficacy. Whether more selective JAK inhibition, including gut restrictive JAK inhibition, will result in similar efficacy with better safety data than tofacitinib has to be awaited.

Lacking head-to-head clinical trials or comparative effectiveness studies with tofacitinib, only indirect evidence from network meta-analyses and real-life studies can currently be used to position tofacitinib. A recent network meta-analysis did rank infliximab highest in biological-naive UC patients, whereas ustekinumab and tofacitinib were ranked highest in anti-tumour necrosis factor (TNF) exposed UC patients.^4^ Results of this meta-analysis should be interpreted with caution because re-random allocation of induction responders in phase III maintenance trials might introduce selection bias. Therefore, head-to-head clinical trials, designed and powered to compare distinct therapies or therapeutic strategies and based on an intention-to-treat analysis, are needed to determine the ranking of new treatments.

Although head-to-head trials will be informative on a population level, they may provide less guidance for an individual patient as long as biomarkers are not yet incorporated into head-to-head performance trials. Hence, to determine the exact place of tofacitinib – and by extent all available compounds – in treatment algorithms, predictive (bio)markers are eagerly awaited. Collaborative efforts, ideally between academia and pharma, collecting detailed patient characteristics (phenomics) and several other molecular layers (genomics, transcriptomics, metabolomics, proteomics, metagenomics) aiming to unravel disease heterogeneity and identify predictive markers, should be largely encouraged, both during and after drug development.

Besides suggestions coming from the OCTAVE programme and network meta-analyses,^4,5^ real-life prospective evidence is needed to provide clinical outcomes of tofacitinib in patients not represented in phase III trials (e.g. prior to anti-adhesion or anti-interleukin use, history of malignancy, pregnancy).^6^ Applied treatment strategies in daily clinical practice should be analysed in post-marketing registries to explore the unique capabilities of tofacitinib. Due to its rapid onset of action and quick drug clearance, high-intensity tofacitinib has, for instance, been suggested in acute severe colitis as rescue therapy after steroid or infliximab failure.^7^ However, given the increased infectious risk in patients with lymphopenia and/or high dose concomitant corticosteroids combined with JAK-inhibitors,^8^ more data, ideally randomised, are warranted before considering tofacitinib as standard of care in acute severe UC.

Does tofacitinib still have a role in the treatment algorithm of Crohn’s disease (CD), as drug development was terminated in phase II? Despite not having met the primary and secondary endpoints of the study,^9^ a modest – although not significant – dose-related reduction in inflammatory biomarkers was observed at week 4, suggesting a mild biological effect. Several explanations for the negative trial could be raised, including the high placebo rates, the lack of central reading and a clinically defined primary endpoint instead of an objective marker of intestinal inflammation. Hence, real-world data in (highly) refractory CD patients treated with off-label tofacitinib have been collected, reporting some effect after 8 weeks of treatment.^10^ As more selective JAK inhibitors (filgotinib, upadacitinib, TD-1473, PF-06651600, PF-06700841) are currently showing promising results in (late stage) drug development for CD,^3^ the role of tofacitinib in CD will be limited with only some off-label use in highly refractory patients, without guarantee of therapeutic success.

In conclusion, tofacitinib is a promising, first-in-class treatment option for patients with UC, whereas its added value in CD is very limited. Due to its unique characteristics, including its oral administration and lack of immunogenicity, it offers certain benefits compared with biological agents in UC. However, safety concerns remain an important limitation, especially with high dosing, which needs further study. Because of the rapidly expanding therapeutic armamentarium in UC, head-to-head trials including predictive biomarkers are urgently needed to redefine current treatment algorithms.
